# Association of IL-10–592 C > A /-1082 A > G and the TNFα -308 G > A with susceptibility to COVID-19 and clinical outcomes

**DOI:** 10.1186/s12920-023-01793-4

**Published:** 2024-01-29

**Authors:** Raghda E. Eldesouki, Rania M. Kishk, Noha M. Abd El-Fadeal, Rama I Mahran, Noha Kamel, Eman Riad, Nader Nemr, Safaa M. Kishk, Eman Abdel-Moemen Mohammed

**Affiliations:** 1 Genetics Unit, Histology Department, Faculty of Medicine, Suez Canal University, 41522 Ismailia, Egypt; 2 Microbiology and immunology Department, Faculty of Medicine, Suez Canal University, Ismaila, Egypt; 3 Medical Biochemistry and Molecular Biology Department, Faculty of Medicine, Suez Canal University, Ismaila, Egypt; 4 Biochemistry Department, Ibn Sina National College for Medical Studies, Jeddah, Kingdom of Saudi Arabia; 5 Clinical Pharmacology Department, Faculty of Medicine, Suez Canal University, Ismaila, Egypt; 6 Clinical Pathology Department, Faculty of Medicine, Suez Canal University, Ismaila, Egypt; 7 Pulmonology Unit, Internal Medicine Department, Faculty of Medicine, Suez Canal University, Ismaila, Egypt; 8 Endemic and Infectious Diseases Department, Faculty of Medicine, Suez Canal University, Ismailia, Egypt; 9 Pharmaceutical Medicinal Chemistry Department, Faculty of Pharmacy, Suez Canal University, Ismailia, Egypt

**Keywords:** SARS-CoV-2, *IL-10*, TNFa, *rs1800872 (− 592)*, *rs1800896(− 1082)*, *rs1800629(-308)*, Innate immunity, COVID-19, Polymorphism, SNP, Genotype, Pharmacogenomics, Remdesivir, Egypt

## Abstract

**Background:**

Variation in host immune responses to SARS-CoV-2 is regulated by multiple genes involved in innate viral response and cytokine storm emergence like *IL-10* and *TNFa* gene polymorphisms. We hypothesize that *IL-10*; *-592 C > A* and *− 1082 A > G* and *TNFa-308 G > A* are associated with the risk of SARS-COV2 infections and clinical outcome.

**Methods:**

Genotyping, laboratory and radiological investigations were done to 110 COVID-19 patients and 110 healthy subjects, in Ismailia, Egypt.

**Results:**

A significant association between the − 592 A allele, A containing genotypes under all models (*p* < 0.0001), and *TNFa* A allele with risk to infection was observed but not with the G allele of the − 1082. The − 592 /-1082 CG and the − 592 /-1082/ -308 CGG haplotypes showed higher odds in COVID-19 patients. Severe lung affection was negatively associated with − 592, while positive association was observed with − 1082. Higher D-dimer levels were strongly associated with the − 1082 GG genotype. Survival outcomes were strongly associated with the GA genotype of *TNFa*. -308 as well as AGG and AAA haplotypes.

**Conclusion:**

*IL-10* and *TNFa* polymorphisms should be considered for clinical and epidemiological evaluation of COVID-19 patients.

**Supplementary Information:**

The online version contains supplementary material available at 10.1186/s12920-023-01793-4.

## Introduction

Coronavirus disease 2019 (COVID-19) is the newly emergent acute respiratory distress syndrome (ARDS) caused by the severe acute respiratory syndrome coronavirus 2 (SARS-CoV-2) that elicits a cytokine storm [1]. A cytokine storm is an aberrant systemic inflammatory response caused by an uncontrolled overproduction of inflammatory cytokines [2, 3]. If no appropriate treatment is given to patients suffering from a cytokine storm, acute lung damage progresses to ARDS accompanied by multi-organ failure, followed by death [4]. The development of COVID-19-associated ARDS (CARDS) has shown inter-individual differences in terms of risk and clinical outcomes. Plasma cytokine levels were found to be associated with the clinical severity of CARDS [5, 6, 7, 8–10]. Pharmacogenetic variations have been shown to affect the response of COVID-19 patients as well [11]. Polymorphisms in genes related to pulmonary protection, innate immune response, and inflammation have been reported to be associated with clinical outcomes in SARS-CoV-2 [5, 12–15] infections other than those related to ARDS [16, 17]. More attention was brought to studying the interaction between *IL-10* alleles with the tumor necrotic factor alpha (TNFa) alleles, for their opposing roles [18, 19]. Three polymorphisms; the *IL-10*; the *− 592 C > A (rs1800872)* and *IL-10* -*1082 A > G (rs1800896)* and the *TNFa; -308 G > A (rs1800629)* were found to correlate with susceptibility and clinical progress of different viral infections [18, 20–23] and as predictors for severity and mortality in patients with COVID-19 [3]. The *− 592 C > A* and *− 1082 A > G* polymorphisms of the IL-10 gene lie within a negative regulatory region that binds to ETS and STAT-3 transcription factors, respectively [24, 25] while the *TNFa − 308 G > A* polymorphism is a regulatory variant that functionally impacts TNFa expression depending on the type of stimulus and the cell type [24]. Different populations have been evaluated for the impact of these polymorphisms [12, 13] on clinical outcomes and severity yet no studies were conducted in Egyptian populations. Thus, we were interested in investigating the association between the same polymorphisms and susceptibility to COVID-19 infection, clinical outcomes and response to remdisivir, in an Egyptian cohort.

## Materials and methods

### Ethical considerations

The study was approved by the Ethics Committee of the School of Medicine Suez Canal University (#4425). It was conducted in compliance with the ethical principles of the Helsinki Declaration for medical research involving human subjects. All participants provided written informed consent.

### Specimens and patient data

One hundred and ten inpatients with COVID-19 and 110 healthy controls were included. Samples were collected through the time frame between April and July 2021, from the Suez Canal Medical School Hospitals. Sample size was calculated using the following equation: n= [(Zα/2)/E]^2^ *P(1-p),where: n = sample size, Zα/2 = 1.96 (The critical value that divides the central 95% of the Z distribution from the 5% in the tail), P = the prevalence of SARS-CoV2 = 0.1% (as confirmed by the WHO for December 2020 divided by the Egyptian population in December 2020) (https://covid19.who.int/region/emro/country/eg). E = Margin of error (= Width of confidence interval) = 5%. Patients of both genders were randomly selected.

COVID-19 was diagnosed based on a chest computed tomography (CT) scan (multiple bilateral ground-glass opacities mainly in the periphery), then nasopharyngeal specimens were confirmed for SARS-CoV-2, using molecular PCR assay (Viasure, CerTest Biotec) [26]. For COVID-19 patients, inclusion criteria included a confirmed diagnosis. Controls were documented as COVID-19 negative, matched for age, gender, and demographics, and free from severe respiratory conditions. Exclusion criteria for controls involve a history of COVID-19 and chronic diseases unless of specific interest.

### Clinical data

Clinical data were collected from patients’ records. Symptom-onset date and comorbidities (hypertension, diabetes, chronic kidney disease (CKD), coronary heart disease (CHD), or chronic liver disease (CLD)) were collected using a questionnaire filled by the physician at patient’s admission. At the time of diagnosis, all COVID-19 patients had routine tests such as a complete blood count, C-reactive protein (CRP), D-dimer, serum ferritin, and lactate dehydrogenase (LDH) [27] and severity was classified as severe versus non-severe based on cut-off values [28]. Patients with pulmonary infiltrates above 50% on CT of the lung were considered severely affected. The need for high flow oxygen therapy; > 40% (by nasal cannula, oxygen face mask, or venturi mask, or continuous positive airway pressure via non-invasive CPAP) was used to determine the severity of respiratory symptoms [29]. The pO2 levels of all patients was monitored during their stay in the hospital.

### Antiviral treatment

To investigate the relationship between the response of patients to remdesivir treatment and the polymorphisms, we studied the response of patients to remdesivir treatment (*n* = 30) in different polymorphisms and compared to non remdesivir treated patients (*n* = 81) in regard to CT picture, laboratory findings, and survival. The data of other drugs that were prescribed to the patients such as tocilizumab, antimicrobials, corticosteroids, anticoagulants, were also collected.

### Genotyping assays

Peripheral blood was collected from each patient and DNA was extracted using the GeneJET Whole Blood Genomic DNA Purification Minikit (Thermo Scientific, inc) according to the manufacturer’s instructions. Genotyping was done using TaqMan SNP assays for Interleukin-10 SNPs; *rs1800872*(ID_ C___1747363_10), *rs1800896* (ID_ C___1747360_10 ) and TNFa SNP *rs1800629*(ID_ C___7514879_10) (Applied Biosystems, Foster City, CA).

### Statistical analysis

Different genotypes of *IL10* and *TNFa* were compared in terms of disease severity, laboratory findings, response to treatment, and final outcome after genotyping. IBM SPSS 2013 was used to analyze the data. SNP stat, was used to identify polymorphisms. Using Hardy-Weinberg equilibrium, we compared group genotype distributions to the population’s overall genotype distributions. The significance of the obtained results was set at 0.05. To calculate the percent of survival in Remdesivir treated versus the non remdesivir- treated patients in different polymorphisms, the number of the patients survived after treatment with or without remdesivir treatment in each polymorphism was divided by the total number of patients in each polymorphism in each group and this number was multiplied by 100. The significance of the percentage of patient survival among the different alleles in each polymorphism and in the remdesivir treated group versus the non remdesivir treated was analyzed by ANOVA.

## Results

### Characteristics of studied groups and genotype distribution within COVID-19 patients

This way a case-control study including 110 COVID-19 patients matched with age and gender to 110 healthy individuals. The patients’ group had comorbidities like diabetes, hypertension, heart diseases, chronic liver diseases, and chronic kidney diseases while individuals within the control group were completely free (Table S1).

The mean age of the studied patients was 57 ± 15.2 in cases versus 56.6 ± 14.5 in controls. PCR positive patients represented 80% of cases, and a significant positive association was only observed between positive PCR and *TNFa rs1800629* genotypes (*p* < 0.05). A significant difference was observed with the genotypes of TNFa polymorphism and hypertension (*p* = 0.04) (Table 1). On comparing different genotypes with laboratory results, no significant difference was observed between groups except for the GG versus the GA genotypes of the *IL-10* gene polymorphism; *rs1800896 (− 1082)*, where a significant difference was observed between low lymphocyte count and increased CRP (*p* < 0.05) (Table 1).

**Table 1:** Demographic, clinical characteristics and laboratory parameters of COVID-19 patients and healthy subject*

SNP_ID	IL10 rs1800872 (− 592)					IL10 rs1800896 (− 1082)					TNFa rs1800629 (-308)		
Genotypes/Variables	AA	CA	CC	*p* value	*p* value**	AA	AG	GG	*p* value	*p* value**	AG	GG	*p* value
**Age (years, Mean ± SD)**	56.2 ± 16.3	58.4 ± 13.2	50.1 ± 22.9	0.4	P1 = 1 P2 = 1 P3 = 0.6	57.4 ± 15.1	51.5 ± 13.6	59.5 ± 16.3	0.23	P4 = 0.4 P5 = 1 P6 = 0.2	56.3 ± 16.1	57.1 ± 15.1	0.89
**Sex**													
**Male**	25	30	2	0.6	-	33	8	16	0.52		14	43	0.19
**Female**	23	25	5		-	35	9	9			7	46	
**Diabetes Mellitus**													
**Positive**	22	25	2	0.8	-	28	10	13	0.39	-	7	42	0.34
**Negative**	27	30	4		-	41	7	11		-	13	48	
**Hypertension**													
**Positive**	19	23	1	0.4	-	28	7	8	0.8	-	4	39	0.04
**Negative**	30	32	5		-	41	10	16		-	16	51	
**CKD**					-					-			
**Positive**	1	0	1	0.015	-	2	0	0	0.54	-	0	2	0.50
**Negative**	48	55	5		-	67	17	24		-	20	88	
**PCR test**	47	56	7			68	17	25			20	90	
**Positive**	38(43%)	44	7	0.3965		52	14	23	0.2368		13	76	0.04536
**Negative**	9(19%)	12	0			16	3	2			7	14	
**White blood cell count (/µl)**	11,020 ± 0.04	11,091 ± 0.04	10,000 ± 0.001	0.7	P1 = 1 P2 = 1 P3 = 1	1.1159 ± 0.03	1.0588 ± 0.05	1.0833 ± 0.05	0.75	P4 = 1 P5 = 1 P6-1	11,000 ± 0.06	11,000 ± 0.03	1.0
**Lymphocytes (/µl)**	1820.3 ± 618.9	11399.1 ± 200.3	1197 ± 259.8	0.7	P1 = 1 P2 = 1 P3 = 1	1129.8 ± 85.1	3845.4 ± 1802.1	1249.9 ± 110.2	0.86	P4 = 0.003 P5 = 1 P6 = 0.019	1139.7 ± 126.4	1672.6 ± 356.3	0.48
**C reactive protein (mg/l)**	81.3 ± 9.3	63.6 ± 9.1	66.4 ± 26.8	0.3	P1 = 0.5 P2 = 1 P3 = 1	73.7 ± 7.1	32.2 ± 5.7	93.5 ± 19.1	0.19	P4 = 0.05 P5 = 0.5 P6 = 0.01	69.4 ± 14.6	72.1 ± 7	0.87
**Ferritin (ng/ml)**	561.7 ± 72.03	476.9 ± 66.6	552.1 ± 134.2	0.6	P1 = 1 P2 = 1 P3 = 1	556.2 ± 61.1	352.5 ± 73.9	529 ± 110.1	0.81	P4 = 0.3 P5 = 1 P6 = 0.7	491.3 ± 93	523.3 ± 53.2	0.79
**D-Dimer (ng/ml)**	1282.4 ± 295.2	752.8 ± 138.1	1639 ± 731	0.1	P1 = 1 P2 = 0.2 P3 = 0.6	983.2 ± 592.4	755.5 ± 272.4	1520.8 ± 463.2	0.13	P4 = 1 P5 = 0.3 P6 = 0.4	612.6 ± 111.3	1131.4 ± 168.3	0.19
**LDH (U/l)**	423.3 ± 41.7	401.9 ± 45.8	330.3 ± 52.3	0.7	P1 = 1 P2 = 1 P3 = 1	415.3 ± 40.5	307.1 ± 39.8	458.1 ± 61.1	0.56	P4 = 0.5 P5 = 1 P6 = 0.3	408.8 ± 47.5	407.1 ± 34.6	0.98

*Demographic data were analyzed for COVID-19 patients (*n* = 110) and healthy subjects (*n* = 110), while laboratory and clinical characteristics were analyzed within the COVID-19 patients group (*n* = 110). **: Within group P1: difference between AA and CA, P2: difference between AA and CC, P3: difference between CA & CC. P4: difference between AA & AG, P5: difference between AA & GG, P6: difference between GG & AG Parameters described as mean ± SEM LDH: Lactate Dehydrogenase

### Alleles/Genotype frequencies and distribution of *IL10* and *TNFa* polymorphisms under different inheritance models

The genotype distribution among the patients versus the control groups are shown in Table 2. The AA genotype of *IL-10 (-592)* (*rs1800872)* showed significantly higher frequency among the patients compared to the control group *(p* < 0.001). Hardy–Weinberg equilibrium was found among controls but not among the cases. Under all models, the mutant A allele frequency was higher in COVID − 19 patients than in healthy individuals (Table 3).

**Table 2:** Genotype distribution of *IL-10* and *TNFa* polymorphisms in COVID-19 patients versus healthy subjects

Gene	SNP_ID	Alleles and Genotype	Cases *n* = 110 (%)	Controls *n* = 110(%)	Adjusted OR* (95% CI)	*p* value**
**IL-10**	rs1800872 (− 592)	A	150(68)	92(42)	Reference	
		C	70 (32)	128(58)	2.9814 (2.0181 to 4.4043)	*p* < 0.0001
		CC	7(6)	24(22)	Reference	
		CA	56(51)	80(73)	0.0734(0.0291 to 0.1849)	*p* < 0.0001
		AA	47(43)	6(5)	0.0372 (0.0113 to 0.1232)	*p* < 0.0001
**X** **2** **HWE** *** (***p***-**value**)			0.083	< 0.0001		
***IL-10***	rs1800896 (− 1082)	A	152(70)	136(62)	Reference	
		G	64(30)	84(38)	0.6817(0.4574 to 1.0161)	*p* = 0.0599
		AA	68(63)	37(34)	Reference	
		GA	16(15)	62(56)	0.1404(0.0711 to 0.2772)	*p* = 0.0599
		GG	24(22)	11(10)	1.1872(0.5238 to 2.6909)	*p* = 0.6811
**X2 HWE** (***p***-**value**)			< 0.0001	0.068		
***TNFa***	rs1800629 (-308)	G	200(91)	220(100)	Reference	-
		A	20(9)	0	45.0898 (2.7093 to 750.4146)	*p* = 0.0079
		GG	90 (81.8)	110 (100)	Reference	
		GA	20 (18.2)	0 (0)	50.0608(2.9863 to 839.1787)	*p* = 0.0065
		AA	0	0	1.2210(0.0240 to 62.1470)	*p* = 0.9207
**X2 HWE** (***p*** **value)**			0.6	1		

CI, Confidence interval; *IL-10*: Interleukin 10, *TNFa*: Tummor necrotic factor alpha; *OR, odds ratio

**p value *<* 0.05 was considered as significant. Age, gender and comorbid diseases were considered as confounding variables to derive the adjusted odds ratio. ***X2HWE: Hardy Weinberg Equilibrium using Chisquare

For the second *IL-10* polymorphism, *(− 1082) (rs1800896)*, a higher frequency of the wild type A (70%) allele and AA genotype (63%) in patients as compared to healthy individuals, was observed (Table 2). Hardy–Weinberg equilibrium was only observed in cases (*p* < 0.001). Under multiple models, the G allele and GA genotypes were associated with a lower risk of developing COVID-19 disease (Table 3). *TNFa* polymorphism was tested, and only two genotypes were detected: the AG and GG. The wild-type allele G was present in 100% of healthy individuals and 91% of patients (*p* = 0.0079). The GA genotype was more prevalent in patients than in healthy subjects (*p* = 0.0065, OR = 50.0608, CI = 2.9863 to 839.1787) (Table 2).

**Table 3:** Genotype frequencies of *IL-10* studied SNPs in COVID-19 patients versus healthy subjects under different inheritance models

SNP-ID	Model	Genotypes	COVID-19 patients(*n* = 110) (%)	Healthy subjects (*n* = 110) (%)	OR* (95% CI)	*p*- value**
***rs1800872***	Co dominant	AA	46(42.2)	6 (5.5)	1.00	
		CA	56(51.4)	80 (72.7)	11.20 (4.44–28.23)	< 0.0001
		CC	7(6.4)	24 (21.8)	25.93 (7.76–86.60)	
	Dominant	AA	46 (42.2%)	6 (5.5%)	1.00	
		CA + CC	63 (57.8%)	104 (94.5%)	12.89 (5.17–32.16)	< 0.0001
	Recessive	AA + CA	102 (93.6)	86 (78.2%)	1.00	
		CC	7 (6.4)	24 (21.8%)	3.95 (1.61–9.68)	< 0.0001
	Over Dominant	AA + CC	53 (48.6)	30 (27.3)	1.00	
		CA	56 (51.4)	80 (72.7)	2.60 (1.47–4.60)	< 0.0001
	Log-additive	---	---	---	5.68 (3.09–10.43)	< 0.0001
***rs1800896***	Co dominant	AA	68 (63%)	37 (33.6%)	1.00	
		GA	16 (14.8%)	62 (56.4%)	7.12 (3.61–14.06)	< 0.0001
		GG	24 (22.2%)	11 (10%)	0.84 (0.37–1.91)	
	Dominant	AA	68 (63%)	37 (33.6%)	1.00	
		GA + GG	40 (37%)	73 (66.4%)	3.35 (1.92–5.85)	< 0.0001
	Recessive	AA + GA	84 (77.8%)	99 (90%)	1.00	
		GG	24 (22.2%)	11 (10%)	0.39 (0.18–0.84)	0.013
	Over Dominant	AA + GG	92 (85.2%)	48 (43.6%)	1.00	
		GA	16 (14.8%)	62 (56.4%)	7.43 (3.87–14.24)	< 0.0001
	Log-additive		---	---	1.38 (0.95–1.99)	0.085

*OR, odds ratio., CI, Confidence interval, **p value *<* 0.05 was considered as significant. Age and gender were considered as confounding variables to derive the adjusted odds ratio

### Haplotype distribution of *IL-10* and *TNFa* polymorphisms

Further haplotype association analysis was done for the genetic polymorphisms under investigation, and the risk of developing COVID-19 (Table 4). The haplotypes for the *IL-10* polymorphisms showed that the CG haplotype was more frequent in COVID-19 patients (*p* < 0.0001, OR = 16.97, CI = 6.36–45.29). Then, the *TNFa* polymorphism haplotype was evaluated along with the *IL-10* alleles. The AAG was the prevalent haplotype in healthy individuals as compared to patients with COVID-19, with CGG haplotype showing higher frequency in COVID-19 patients (*p* < 0.0001, OR = 14.96, CI = 5.55–40.32). Other haplotypes did not show significant associations (Table 4).

**Table 4:** Haplotype frequencies and distribution of *IL-10* and *TNFa* in COVID-19 patients versus healthy subjects (*n* = 220)

IL-10 (-592)	IL-10 (-1082)	Frequency	OR (95% CI)	*p*-value
A	A	0.4456	1.00	---
C	G	0.2388	**16.97 (6.36–45.29)**	< 0.0001
C	A	0.2112	1.72 (0.84–3.54)	0.14
A	G	0.1044	0.00 (-Inf - Inf)	1
***IL-10*** **haplotypes**	***TNFa*** (-308)	**Frequency**	**OR (95% CI)**	***p*** **-value**
H1(AA)	G	0.4277	1.00	---
H2(CG)	G	0.2398	**14.07 (5.28–37.50)**	< 0.0001
H3(CA)	G	0.2004	1.72 (0.82–3.59)	0.15
H4(AG)	G	0.0866	0.00 (-Inf - Inf)	1

OR, un-adjusted odds ratio, CI, Confidence interval, *p* value *<* 0.05 was considered as significant. Global haplotype association p-value: <0.0001. Haplotypes for the IL-10 polymorphisms are in the first part. No significant OR were identified with TNF1 A allele

### Association of inheritance models of *IL-10* and *TNFa* SNPs with clinical outcomes in patients with COVID-19 disease

Being involved in cytokine storm [30], we evaluated the association of genotypes with the serum ferritin and D-dimer results in addition to the radiological findings, and oxygen support. Table 5 shows the distribution of different models of inheritance and odds ratios were calculated between severe versus non-severe cases.

**Table 5:** Association of inheritance models of IL-10 and TNFa SNPs with clinical and laboratory severity*

SNP_ID	Model	Genotype	OR(CI) (p value)			
			O2 therapy	HRCT	Ferritin	D-Dimer
***rs1800872***	**Co dominant**	AA	1.00	1.00	1.00	1.00
		CA	0.95 (0.41–2.21) (0.98)	0.47 (0.20–1.10) (0.12)	0.62 (0.19–2.06) (0.37)	1.10 (0.49–2.49) (0.91)
		CC	0.85 (0.15–4.91)	0.25 (0.03–2.21)	1.90 (0.50–7.27)	1.42 (0.25–8.11)
	**Dominant**	AA	1.00	1.00	1.00	1.00
		CA + CC	0.94 (0.42–2.13) (0.89)	0.44 (0.19–1.01) (0.051)	1.10 (0.42–2.90) (0.84)	1.13 (0.51–2.50) (0.76)
	**Recessive**	AA + CA	1.00	1.00	1.00	1.00
		CC	0.88 (0.16–4.75) (0.88)	0.36 (0.04–3.11) (0.3)	2.11 (0.57–7.82) (0.23)	1.34 (0.25–7.27) (0.73)
	**Over dominant**	AA + CC	1.00	1.00	1.00	1.00
		CA	0.97 (0.43–2.19) (0.95)	0.54 (0.23–1.24) (0.14)	0.54 (0.17–1.73) (0.31)	1.06 (0.48–2.32) (0.89)
	**Log additive**		0.94 (0.48–1.85) (0.86)	**0.48 (0.23–0.99)** **(0.04)**	1.23 (0.68–2.22) (0.49)	1.14 (0.59–2.21) (0.69)
***rs1800896***	**Co dominant**	AA	1.00	1.00	1.00	1.00
		GA	2.02 (0.66–6.14) (0.15)	1.87 (0.59–5.97) (0.13)	0.47 (0.14–1.66) (0.32)	0.94 (0.32–2.77) (0.066)
		GG	2.44 (0.92–6.46)	2.64 (0.99–7.05) (0.13)	1.62 (0.41–6.36) (0.32)	3.46 (1.07–11.19) (0.066)
	**Dominant**	AA	1.00	1.00	1.00	1.00
		GA + GG	2.26 (0.99–5.16) (0.053)	2.31 (0.99–5.36) (0.051)	0.90 (0.33–2.46) (0.84)	1.86 (0.80–4.31) (0.14)
	**Recessive**	AA + GA	1.00	1.00	1.00	1.00
		GG	2.08 (0.82–5.29) 0.13	2.31 (0.90–5.91) (0.084)	1.91 (0.50–7.21) (0.32)	**3.50 (1.10–11.10)** **(0.02)**
	**Over dominant**	AA + GG	1.00	1.00	1.00	1.00
		GA	1.55 (0.53–4.49) 0.42	1.40 (0.46–4.24) (0.56)	0.42 (0.12–1.43) 0.18)	0.71 (0.25–2.06) (0.54)
	**Log additive**			**1.64 (1.01–2.66)** **(0.45)**	1.12 (0.61–2.04) (0.71)	1.63 (0.96–2.76) (0.06)
***rs1800629***	**---**	GG	1.00	1.00	1.00	1.00
	**---**	AG	1.56 (0.57–4.24) (0.39)	0.73 (0.24–2.20) (0.56)	1.09 (0.32–3.73) (0.89)	1.29 (0.45–3.68) (0.63)

*HRCT: high resolution compute topography. OR, odds ratio adjusted to Gender and age. Severe versus non-severe odds were compared. CI, Confidence interval, *p* value *<* 0.05 was considered as significant

Under the additive inheritance model, the *IL-10* polymorphisms (− 1082) showed non-significant association with severe HCRT finding (*p* = 0.45, OR = 1.64, CI = 1.01–2.66) while severe COVI-19, based on D-dimer levels, was associated with the recessive model (*p* = 0.02, OR = 3.5, CI = 1.10–11.0). Although the *TNFa* polymorphism (-308) didn’t show any association with clinical and laboratory severity findings, the AG genotype was associated with favorable outcomes and patients’ survival on discharge (*p* = 0.024, OR = 3.3(1.16–9.54)4.29, CI = 1.2–15.3), unlike the *IL-10* polymorphisms (Table 6).

**Table 6:** Association between *IL-10* and *TNFa* SNPs with condition at discharge

SNP_ID	Model	Genotypes	Survived (*n* = 87) (%)	Deceased (*n* = 23) (%)	OR* (95% CI)	*p*- value**
***rs1800872***	Co dominant	AA	35 (40.2%)	12 (52.2%)	1.00	
		CA	45 (51.7%)	11 (47.8%)	0.67 (0.23–1.98)	0.27
		CC	7 (8.1%)	0 (0%)	0.00 (0.00-NA)	
	Dominant	AA	35 (40.2%)	12 (52.2%)	1.00	0.37
		CA + CC	52 (59.8%)	11 (47.8%)	0.61 (0.21–1.80)	
	Recessive	AA + CA	80 (92%)	23 (100%)	1.00	0.15
		CC	7 (8.1%)	0 (0%)	0.00 (0.00-NA)	
	Over Dominant	AA + CC	42 (48.3%)	12 (52.2%)	1.00	0.68
		CA	45 (51.7%)	11 (47.8%)	0.80 (0.27–2.31)	
	Log-additive	---	---	---	0.55 (0.20–1.47)	0.23
X2 HWE*** (***p*** **value**)			0.23	0.28		
***rs1800896***	Co dominant	AA	57 (65.5%)	11 (47.8%)	1.00	0.42
		GA	13 (14.9%)	4 (17.4%)	0.70 (0.14–3.40)	
		GG	17 (19.5%)	8 (34.8%)	1.94 (0.57–6.58)	
	Dominant	AA	57 (65.5%)	11 (47.8%)	1.00	0.59
		GA + GG	30 (34.5%)	12 (52.2%)	1.35 (0.46–3.97)	
	Recessive	AA + GA	70 (80.5%)	15 (65.2%)	1.00	0.21
		GG	17 (19.5%)	8 (34.8%)	2.11 (0.66–6.76)	
	Over Dominant	AA + GG	74 (85.1%)	19 (82.6%)	1.00	0.42
		GA	13 (14.9%)	4 (17.4%)	0.55 (0.12–2.49)	
	Log-additive	---	---	---	1.35 (0.73–2.50)	0.34
**X2 HWE** (***p*** **value)**	---	---	< 0.0001	< 0.0001		
**rs1800629**	---	GG	75 (86.2%)	15 (65.2%)	1.00	---
	---	AG	12 (13.8%)	8 (34.8%)	**3.3(1.16–9.54)**	**0.024**
**X2 HWE** (***p*** **value)**	---	1	1	-	-	-

*OR, odds ratio adjusted to Gender, Remdisvir, Actmra, anticoagulants, corticosteroids and antibiotic prescription. CI, Confidence interval, *p* value *<* 0.05 was considered as significant

After adjustment of interventional parameters and comorbidities, a strong association with survival outcome was observed with the AGG (*p* = 0.019, OR = 3.92, CI = 1.28–12.04) and the AAA haplotypes (Table 7).

**Table 7:** Haplotype association with survival on discharge

	rs1800872	rs1800896	rs1800629	Freq	OR (95% CI)*	*P*-value	OR (95% CI)**	*P*-value
1	A	A	G	0.4498	1.00	---	1.00	---
2	C	A	G	0.187	1.60 (0.39–6.53)	0.52	1.84 (0.48–7.11)	0.38
3	A	G	G	0.1764	1.83 (0.59–5.65)	0.3	**3.92 (1.28–12.04)**	**0.019**
4	C	G	G	0.0959	0.08 (0.00–3.15)	0.18	0.00 (-Inf - Inf)	1
5	A	A	A	0.0324	**14.25 (1.14–177.79)**	0.042	**24.67 (1.51–401.63)**	**0.027**

*n = 110, adjusted by Gender + Remd + Actmra + Anticog + Corts + Antib, ** adjusted by DM + HTN + IHD + CKD + CLD, p value < 0.05 was considered as significant

 Association with clinical outcomes; whether deceased or survived were analyzed (based on data availability), after adjustment to remdisivir treatment (Table S2). The AG genotype associated with the *TNFa* polymorphism showed protective effect (*p* = 0.022, CI = 0.25 (0.08–0.81).

## Discussion

 The innate immune response is recognized as the primary line of defense against viral infections, including SARS-CoV-2 and MERS. Severe lung damage and elevated mortality rates in these infections are associated with increased levels of both anti-inflammatory and pro-inflammatory cytokines, along with heightened counts of neutrophils and monocyte-macrophages [31, 32]; [33, 34]. Limited studies have delved into the genetic variations of the host innate immune status in COVID-19 patients [12, 13]. o In this study, the focus is on the association between IL-10 and TNFa promoter polymorphisms and susceptibility to clinical outcomes in SARS-CoV-2 infections.The frequencies of IL-10 -592 A and C alleles were 0.55 and 0.45, respectively, resembling the East Asian population but differing from the African population where the C allele predominated over 0.5. IL-10 -1082 A and G allele frequencies were 0.66 and 0.34, respectively, consistent with African populations and global frequencies of 0.7 and 0.3, respectively. TNFa-308 allele frequencies were 0.95 for the G allele and 0.05 for the A allele, resembling some American populations, east and south Asian populations, and the global frequency [24]. Notably, our data were different from Saleh et al. [20] where similar polymorphisms were analyzed in another Egyptian population and G allele of *TNFa-308* frequency didn’t exceed 0.5(0.4), while the A allele was 0.6, which might be explained by their bigger sample size. Our results have also shown that susceptibility to SARS-CoV-2 infection was associated with all the genotypes of *IL-10 -592 C > A* polymorphism, with the C allele favoring protection against infection which was consistent with different viral infections [35]. Additionally, the A-allele was associated with higher *IL-10* serum levels and hence, was considered a predictor of HBV recovery and with HIV susceptibility, especially in the dominant model [36] while the C containing genotypes have shown increased risk for viral infections, in both hetero and homozygote states([37] ([38]) [23, 39].

Interestingly, the GA genotype of *IL-10-1082 A > G* had a significant association with case fatality in influenza A/H1N1pdm09 infections in Indian patients [22, 40] and other viruses [41]. A possible explanation to the association of the *IL-10* polymorphisms, the AA in *IL-10 -592* and the GG in *IL-10 -1082*, with viral infection susceptibility/persistence, is that the increase in IL-10 serum levels exerts sustained inhibitory effects on immune cells that are responsible for viral clearance [42]. On the contrary, no association was observed between the *IL-10* polymorphisms and the Mediterranean spotted fever [43] dengue virus [44, 45] and hepatitis sustained viral responses [46, 47]. Our results of diminished AA genotype of the *TNFa − 308G > A* polymorphism were consistent with other studies on different populations such as the Sicilian patients (1.2%) [43] and the Chinese patients (1%) [47], but not with another Egyptian population where the AA genotype had a prominent presence among patients (32.0%) and was associated with an aggressive disease outcome [20]. A significantly higher frequency of *TNFa − 308G > A* polymorphism genotype was associated with severity in Influenza A/H1N1pdm09 in Indian patients [22]. We show that the G allele confers protection to symptomatic COVID-19 in contrary to dengue virus where the A allele was protective. While the GA genotype of *TNFa − 308* was protective against dengue virus infection, it was significantly associated with COVID-19 infection. In addition, the A allele of *TNFa − 308G > A* was protective against TTV [23]. In this study, CRP, leucocyte and lymphocytes count, D-dimer, ferritin levels, and LDH levels were tested. High levels of CRP and low lymphocytes in COVID-19 patients showed significant differences between the different *IL-10 -1082 A > G* polymorphisms, specifically between AG and GG genotypes (*p* < 0.05) which was consistent with other studies on the effect of these polymorphisms on the CRP levels land lymphocytes’ counts in viral-induced encephalitis [37]. Noteworthy, the risk of venous thrombosis was found to be associated with the *IL-10* promoter polymorphisms; *-592* and *− 1059*, through down-regulation to tissue factor (TF) expression [48]. This is consistent with our results where, the *IL-10-1082* GG genotype is significantly associated with higher hypercoagulability status of the patients, as indicated by D-dimer levels. Studying the effect of IL-10 polymorphisms and *TNFa* on the risk of ARDS, showed inconsistent results. The *IL-10 -1082* GG genotype was significantly associated with an increased ARDS odds (< 52 years of age) [49]. This was consistent with our data where a significant association of the additive model of *IL-10 -1082*, with severe HRCT results was observed. We found that there is no association between the *IL-10 -509* polymorphism and the ARDS development. Similarly, there is currently no reported association between *IL-10-509* polymorphisms and the ARDS development. There was no association between *IL-10* and *TNFa* polymorphisms and interstitial lung fibrosis [27]. Our results showed a significant association between COVID-19 and either the *− 592/-1082* (CG) haplotype or the *− 592/-1082/-308*(CGG) haplotypes, which was similar to CG disease association in Caucasians [49] Not only this, but also, we found a strong association between the *− 592/-1082/-308* AGG and AAA haplotypes and favorable outcomes. A similar risk has been reported between the IL-10 AA haplotypes and the outcome of EBV [42]. The SARS-CoV-2 antiviral treatment; Remdivir, has been used for its anti-inflammatory effect. Although better survival outcomes were associated with remdesivir treatment. One limitation in this study, especially for the investigation to the response of the patients to remdesivir therapy, is the small sample size for the patients on remdesivir treatment. This is because remdesivir is not given as a routine therapy for all COVID-19 patients, but it is reserved to those who are severely ill. However, the normal distribution of the data between the treated and the non-treated group allowed for the comparison between the 2 groups. Deviation from Hardy-Weinberg equilibrium is another limitation that can be justified by considering factors such as selection pressure, population substructure, migration, mutation, genetic drift, and genotyping errors. Bigger sample size and more robust genotyping is recommended to validate our results. Elaborated studies on other cytokines, IL-6 and INF gamma, and their correlation/association to COVID-19 clinical outcomes need to be done. Our data is the first to unravel the association between polymorphisms in *IL-10* gene promoter and COVID-19 in an Egyptian population, which gives insight into the genetic diversity of immune responses towards SARS-COV2. We provided complementary evidence to the role of *TNFa-308* polymorphism in a different Egyptian population [20] and were consistent with findings in Iranian population [50]. In conclusion, this study sheds light on the genetic determinants influencireatment response, and clinical outcomes in SARS-CoV-2 infection. The identification of specific polymorphisms in genes related to inflammatory responses not only allows for risk stratification and personalized treatment approaches but also aids in predicting disease severity and assessing survival outcomes. These findings provide a foundation for individualized patient management, with the potential to optimize resource allocation, enhance monitoring strategies, and improve overall clinical decision-making. While these genetic insights offer valuable contributions to the understanding of COVID-19, further research and validation are imperative for the translation of these findings into routine clinical practice, ensuring their applicability across diverse populations.

### Electronic supplementary material

Below is the link to the electronic supplementary material.

Supplementary Material 1

## Data Availability

All data generated or analyzed during this study are included in this published article (and its supplementary information files).
