# Correction to: Association of IL-10-592 C > A /-1082 A > G and the TNFα -308 G > A with susceptibility to COVID-19 and clinical outcomes

**DOI:** 10.1186/s12920-024-01834-6

**Published:** 2024-02-26

**Authors:** Raghda E. Eldesouki, Rania M. Kishk, Noha M. Abd El-Fadeal, Rama I Mahran, Noha Kamel, Eman Riad, Nader Nemr, Safaa M. Kishk, Eman Abdel-Moemen Mohammed

**Affiliations:** 1 Genetics Unit, Histology Department, Faculty of Medicine, Suez Canal University, 41522 Ismailia, Egypt; 2 Microbiology and Immunology Department, Faculty of Medicine, Suez Canal University, Ismailia, Egypt; 3 Medical Biochemistry and Molecular Biology Department, Faculty of Medicine, Suez Canal University, Ismailia, Egypt; 4 Biochemistry Department, Ibn Sina National College for Medical Studies, Jeddah, Kingdom of Saudi Arabia; 5 Clinical Pharmacology Department, Faculty of Medicine, Suez Canal University, Ismailia, Egypt; 6 Clinical Pathology Department, Faculty of Medicine, Suez Canal University, Ismailia, Egypt; 7 Pulmonology Unit, Internal Medicine Department, Faculty of Medicine, Suez Canal University, Ismailia, Egypt; 8 Endemic and Infectious Diseases Department, Faculty of Medicine, Suez Canal University, Ismailia, Egypt; 9 Pharmaceutical Medicinal Chemistry Department, Faculty of Pharmacy, Suez Canal University, Ismailia, Egypt

**Correction to: Eldesouki et al. BMC Medical Genomics (2024) 17:40**

10.1186/s12920-023-01793-4

Following the publication of the original article [1], the authors reported errors in the affiliations of some authors due to typesetting errors.

Originally, affiliation 4 was listed as Medical Biochemistry Unit, Ibn sina National College for Medical Studies, Jeddah, Kingdom of Saudi Arabia. It should have written as Biochemistry Department, Ibn Sina National College for Medical Studies, Jeddah, Kingdom of Saudi Arabia.

Furthermore, in the original article, author Rama I Mahran was affiliated with Affiliation 5 that is Clinical Pathology Department, Faculty of Medicine, Suez Canal University, Ismaila, Egypt. The correct affiliation is Clinical Pharmacology Department, Faculty of Medicine, Suez Canal University, Ismailia, Egypt.

In the original article, author Noha Kamel was affiliated with affiliation 6 that is Pulmonology Unit, Internal Medicine Department, Faculty of Medicine, Suez Canal University, Ismaila, Egypt. The correct affiliation is Clinical Pathology Department, Faculty of Medicine, Suez Canal University, Ismailia, Egypt.

Moreover, in the original article, author Eman Riad was affiliated with affiliation 7 that is Endemic and Infectious diseases, Faculty of Medicine, Suez Canal University, Ismaila, Egypt. The correct affiliation is Pulmonology Unit, Internal Medicine Department, Faculty of Medicine, Suez Canal University, Ismailia, Egypt.

In the original version, author Nader Nemr was affiliated with affiliation 8 that is Pharmaceutical Medicinal Chemistry Department, Faculty of Pharmacy, Suez Canal University, 41522 Ismailia, Egypt. The correct affiliation should have been Endemic and Infectious Diseases Department, Faculty of Medicine, Suez Canal University, Ismailia, Egypt.

Meanwhile, in the original article, Author Safaa M. Kishk was affiliated with affiliation 8, that is Pharmaceutical Medicinal Chemistry Department, Faculty of Pharmacy, Suez Canal University, 41,522 Ismailia, Egypt. The correct affiliation is Pharmaceutical Medicinal Chemistry Department, Faculty of Pharmacy, Suez Canal University, Ismailia, Egypt, which is affiliation number 9.

The affiliations presented in this Correction article are the correct version.

The original article [1] has been updated.
