# Supplementary material for: Association of IL-10–592 C > A /-1082 A > G and the TNFα -308 G > A with susceptibility to COVID-19 and clinical outcomes
Source: BMC Med Genomics. 2024 Jan 29;17:40. doi: 10.1186/s12920-023-01793-4 (PMC10826193; doi:10.1186/s12920-023-01793-4)
Supplement: Supplementary file 1 — Supplementary Material 1 [file 12920_2023_1793_MOESM1_ESM.pdf]

Table S1: Demographic characteristics of the studied groups

	**Cases (n=110)**	**Control (n=110)**	***p* value**
**Age (years, Mean ±SD)**	57±15.2	56.6±14.5	0.89
**Sex**			
Male	57	56	0.95
Female	53	54	
**Diabetes Mellitus**			
Positive	49	0	0.0001*
Negative	61	110	
**Hypertension**			
Positive	43	0	0.0001*
Negative	67	110	
**Comorbidities**			
**Heart Disease**	10	0	0.002*
**CLD**	7	0	0.01*
**CKD**	2	0	0.49

Student t-test, $\chi 2$: Chi-Square test
CLD: chronic liver disease
CKD: Chronic kidney disease
*P-Value <0.05

Table S2. Clinical outcomes of COVID-19 patients in patients adjusted to remdisivir treatment.

***rs1800872* association with response Discharge (n=109, adjusted by Remd)**							
**Model**	**Genotype**	**Died**	**Survived**	**OR (95% CI)**	***p*-value**	**AIC**	**BIC**
Codominant	AA	12 (54.5%)	35 (40.2%)	1.00	0.12	100.9	111.6
	CA	10 (45.5%)	45 (51.7%)	1.49 (0.54-4.15)			
	CC	0 (0%)	7 (8.1%)	NA (0.00-NA)			
Dominant	AA	12 (54.5%)	35 (40.2%)	1.00	0.26	101.8	109.9
	CA+CC	10 (45.5%)	52 (59.8%)	1.77 (0.65-4.86)			
Recessive	AA+CA	22 (100%)	80 (92%)	1.00	0.057	99.5	107.5
	CC	0 (0%)	7 (8.1%)	NA (0.00-NA)			
Overdominant	AA+CC	12 (54.5%)	42 (48.3%)	1.00	0.71	102.9	111
	CA	10 (45.5%)	45 (51.7%)	1.21 (0.44-3.31)			
Log-additive	---	---	---	1.99 (0.81-4.84)	0.12	100.7	108.7

***rs1800896* association with response Discharge (n=107, adjusted by Remd)**							
**Model**	**Genotype**	**Deceased**	**Survived**	**OR (95% CI)**	***p*-value**	**AIC**	**BIC**
Codominant	AA	11 (47.8%)	55 (65.5%)	1.00	0.35	103.9	114.6
	GA	4 (17.4%)	13 (15.5%)	1.03 (0.25-4.22)			
	GG	8 (34.8%)	16 (19.1%)	0.44 (0.14-1.39)			
Dominant	AA	11 (47.8%)	55 (65.5%)	1.00	0.35	103.1	111.1
	GA+GG	12 (52.2%)	29 (34.5%)	0.62 (0.23-1.69)			
Recessive	AA+GA	15 (65.2%)	68 (81%)	1.00	0.15	101.9	109.9
	GG	8 (34.8%)	16 (19.1%)	0.44 (0.14-1.32)			
Overdominant	AA+GG	19 (82.6%)	71 (84.5%)	1.00	0.65	103.8	111.8
	GA	4 (17.4%)	13 (15.5%)	1.36 (0.35-5.23)			
Log-additive	---	---	---	0.68 (0.38-1.21)	0.19	102.3	110.3

**rs1800629association with response Discharge (n=110, adjusted by Remd)**							
**Model**	**Genotype**	**Deceased**	**Survived**	**OR (95% CI)**	***p*-value**	**AIC**	**BIC**
---	G/G	15 (65.2%)	75 (86.2%)	1.00	0.022	99.4	107.5
	A/G	8 (34.8%)	12 (13.8%)	**0.25 (0.08-0.81)**			
